# A Treatise on Tumours in the Pelvis as a Cause of Difficult Labour; Being the Essay to Which the Prize Was Adjudged by the Medical Faculty of the University of Heidelberg

**Published:** 1841-04

**Authors:** 


### Art. XIV.

*Commentatio de Tumoribus in Pelvi, Partum impedientibus, a gratioso medicorum ordine Heidelbergensi præmio ornata.* Auctore B. R. Puchelt, Med. Chir. et Art. Obstetr. Doctore, cum præfatione F. C. Naegele.—*Heidelbergæ*, 1840. 8vo, pp. 253.

*A Treatise on Tumours in the Pelvis as a cause of difficult labour; being the essay to which the Prize was adjudged by the medical faculty of the University of Heidelberg.* By Dr. B. R. Puchelt. With a Preface by F. C. Naegele, M.D. &c. &c.

This work comes before us with higher claims upon our notice than can be alleged by most prize essays or inaugural dissertations. It is the production of a scholar of Professor Naegele, of Heidelberg, whose recommendatory preface tells us of the diligence, learning, and talent of his pupil; and warns the readers, "ut æqui judices benignam de hoc opusculo ferant sententiam, quippe quæ juvenili auctori præmium sit magnæ, quam impendit, et stimulus incitamentumque futuræ in literis diligentiæ:" nor does the execution of the work discredit this recommendation.

From the great variety of subjects of which the author treats, it will not be possible for us to give anything like a minute analysis of the contents of his book. He divides his essay into two parts; the first treats of tumours of those structures which form the passage for the child, the second of such as are situated in their vicinity, and the whole is preceded by a historical introduction, in which much learning and research are displayed.

These historical notices show how little attention was paid by the early writers to mechanical impediments to labour. The disputes, however, which arose towards the end of the sixteenth century about the propriety of performing the Cæsarean section, led to a more careful examination of those changes in the structure of the pelvis and generative organs which obstruct parturition. Accordingly, Rousset, in his essay on the Cæsarean section, published at Paris in 1581, mentions among the indications for its performance, tumours and other diseases contracting the natural passages; and Arantius, who wrote a few years later, treats expressly, "de mala ossium pelvis conformatione." He was followed by Guillimeau, who detailed a case of labour obstructed by the presence of a calculus in the bladder, and another in which the same result was produced by excrescences about the os uteri. Many of the observations of succeeding writers are to be found in the works of Bonetus, and Stalpart van der Wiel, which were published towards the close of the seventeenth century, and about the same time the treatise of Sylvius de la Boe appeared, "De exostosibus in pelvi partum impedientibus." Since the time of Levret, Smellie, and Roederer, a more correct estimate has been formed of the importance of mechanical impediments to labour, and each succeeding writer has added something to our knowledge of the subject. Dr. Puchelt's task has been to collect and arrange the labours of his predecessors.

In accordance with the author's plan, *tumours of the generative passages* are treated of first. They may be seated either in the *bones* or in the *soft parts*.

1. *In the bones.* They are of two kinds, being either *true exostoses*, or *osteosteatomata;* but the latter are much more unusual than the former. The author details, in chapter i. several cases of exostoses, and next proceeds to draw conclusions with reference to their seat, causes, diagnosis, prognosis, and medical treatment: an arrangement which he follows throughout each subdivision of the work.

It would appear from the cases of exostosis here related, that the inner surface of the os sacrum is their most usual seat; next in frequency the articulation of the last lumbar and first sacral vertebra, and more rarely the body of the last lumbar vertebra. The causes of these tumours are usually very obscure; in two cases, however, the growth of an exostosis was apparently produced by a fall upon the sacrum. Their diagnosis is very difficult; the prognosis is in all cases unfavorable, though of course modified by the size and shape of the tumour. According to the nature of the case, the application of the forceps, perforation of the child's head, or the Cæsarian section may be necessary; but neither the operation of turning, nor the division of the symphysis pubis has led to a good result in those instances in which it was employed.

2. *Tumours of the soft parts.* A. *Tumours of the uterus.* Chapter i. treats of *sarcomatous tumours*; chapter ii. of *steatomatous growths:* both of which are strangely classed among malignant diseases.

Chapter iii. is on *scirrhus and carcinoma of the uterus.* Thirty-two observations are collected by the author, in which labour was complicated with a scirrhous condition of the uterus.

| | cases. |
|---|---|
| "The entire uterus was affected in | 1 |
| The greater portion of the organ | 5 |
| The cervix uteri . . . | 11 |
| The cervix and orifice . . . | 5 |
| The orifice only . . | 6 |
| A portion of the left side . . | 1 |
| A portion of the body . . . | 1 |
| A portion of the fundus . . | 2 |
| | 32." (p. 86.) |

The great danger to which women are exposed by labour thus complicated is shown by the fact that five out of twenty-seven women died in child-birth, and nine very speedily afterwards; while fifteen of the children were born dead. A scirrhous state of the os and cervix uteri is not only the most common form of cancer of the womb, but also by far the most important in obstetric practice, since the management of the labour involves the difficult question, of how far we may trust to nature, or when and how it will be proper to interfere. Even in cases apparently the most unfavorable, the practitioner must not despair of the powers of nature; for several instances are on record in which delivery has been completed without interference, notwithstanding a scirrhous and perfectly rigid state of the os and cervix uteri. In such cases the head of the child has forced its way by distending and gradually lacerating the neck of the womb; and doubts have been raised whether, as a general rule, the fissures made by nature are not to be preferred to incisions with the knife. Madame Lachapelle objects to incising the cervix uteri, but the late M. Baudelocque was an advocate for the practice, which some years since we saw employed with success by Professor Dubois. In fourteen of the cases related by the author, labour was completed by the powers of nature alone, in three the operation of turning was performed, in four the forceps were applied, and in four rupture of the uterus occurred.

In Chapter iv. the author treats of *cauliflower excrescences from the uterus.* The existence of these growths exposes the patient to danger rather from the profuse hæmorrhage to which it gives rise, than from the mechanical obstacle opposed to the passage of the child.

*Fibrous tumours of the uterus* form the subject of the fifth chapter. They are important on account both of their frequency and of the serious hazards to which their existence exposes the patient, not only during labour but also in the puerperal state. In some instances these tumours have acquired so large a size that the bones of the fœtal skull have been fractured during the passage of the child. The powers of the mother become exhausted by the long continuance of labour; and fatal hemorrhage has followed delivery owing to the state of atony in which the uterus has been left by its violent action during labour. Fatal peritonitis is another of the accidents of childbed which these tumours are very prone to induce. Mr. Ingleby's paper in the Edinburgh Medical Journal for January, 1839, seems not to have come under Dr. Puchelt's notice; but the observations there made, "On Fibrous Tumour of the Uterus," will well repay a careful perusal.

Chapter vi. *On polypi, or pediculated fibrous tumours.* From the observations related by the author it would appear that the existence of a polypus in the uterus is a less serious complication of labour than that produced by a fibrous tumour seated in the uterine substance.

Chapter vii. *On encysted tumours.*

Chapter viii. *Prolongation of the anterior lip of the os uteri* sometimes results from the pressure of the head or some other part of the child. Three examples of this occurrence are quoted by the author from an essay which M. Duclos published in the year 1818, in the annals of the medical faculty of Paris; and a fourth case is related which came under the observation of Professor Naegele. The œdematous condition of the os uteri from pressure is the only form of this affection with which the author is acquainted; and, on the authority of a solitary observation of Professor Naegele, he denies that it can offer any impediment to labour, or call for manual interference. In this opinion, however, we can by no means concur, having met with cases in which labour has been seriously impeded by œdema, and other affections of the os uteri, such as are described by Dr. Kennedy in his paper in the Dublin Medical Journal for November, 1838.

*Inflammation*, and *varix of the uterus*, which occupy

Chapters ix. and x. are mentioned as possible causes of obstruction to labour, but no instances are related in which they actually impeded parturition.

B. *Tumours of the vagina* are treated of in six chapters, in which the author follows the same arrangement as in his remarks on tumours of the uterus. We pass over these, however, and come to the second part of the work, wherein are considered *tumours of parts near the generative passages.*

In Chapter i. a single case is related of bony *tumour of the right fallopian tube*, in which the woman died undelivered.

Chapter ii. *On tumours of the ovaries.* The prognosis in these cases is unfavorable, for although the enlarged ovary seldom absolutely prevents delivery, yet a large proportion of women die in childbed. In one instance related by Dr. Puchelt the patient died undelivered; fourteen women died soon after delivery, three at the end of a longer period, and only thirteen permanently recovered.

A difference of opinion exists with regard to the proper management of labour under these circumstances: some, as Davis and Merriman advise trusting much to nature, but Burns warns of the danger of delaying interference. The nature of the assistance to be given is another point upon which authors are by no means agreed. The reposition of the tumour above the brim of the pelvis has been urged by Burns, Baudelocque, and Moreau; Madame Lachapelle agrees in regarding it as desirable, but thinks that the accomplishment of the object would be attended with great difficulty. It has, indeed, been objected that the reposition of the tumefied ovary is likely to be followed by inflammation, suppuration, and death, but in no instance hitherto recorded can the fatal event be clearly referred to any mechanical injury. The evacuation of the contents of the ovary, either by puncture with a trochar or by means of incision, is the second plan which has been proposed. The opening into the tumour may be made either from the vagina or from the rectum: the latter is preferred by Dr. Merriman, who observes that the tumour is usually inclined towards the curvature of the sacrum, and that an incision of any extent into the vagina would be liable to be enlarged by the head of the fœtus in its descent. Lastly, the extirpation of the ovary has been proposed by Merriman, but hitherto has not been practised.

Chapter iii. is on *distention of the rectum*, by the accumulation of fæces.

Chapter iv. treats of the difficulties produced by *over-distention of the bladder*, by the presence of *calculus*, or by a *scirrhous* condition of the bladder.

The next chapter is on *tumours situated in the cellular tissue of the pelvis.* These tumours may be attached to any or all of the bones of the pelvis, and present the same differences in their character as tumours of the uterus. In arranging the numerous observations of these tumours, the author has classified them according to their density, and relates cases of steatomata, scirrhous growths, encysted tumours, and hydatids.

From a comparison of the seventeen observations here recorded, it appears that three women and nine infants died during labour; five women died soon after delivery, seven women and three infants survived, and the fate of one woman and six infants is not mentioned.

The management of these cases must of course vary much according to circumstances. The author gives the following synopsis of the treatment resorted to in the instances he has quoted:

"I. Delivery was accomplished by nature alone in two instances; one of which occurred to Bertrandi, the other to Denman. In both the difficulty was produced by an encysted tumour.

"II Pelletan pressed the tumour into the right side of the pelvis. Both mother and child did well.

"III. Lachapelle emptied the tumour of its contents and turned the child. Mother and child were lost.

"IV. Burns and Drew extirpated the tumour. Both women and one of the children were saved

"V. In a case related by Meier, the forceps were applied without success, and the woman died undelivered. Siebold applied the forceps in another instance, while at the same time the tumour was pressed up above the symphysis pubis. The child was born dead, but the mother recovered.

"VI. The lever was employed in one instance; but Meissner does not state with what result.

"VII. In one case Osiander turned the child which was still-born, but the mother did well. Merriman resorted to the same practice, but lost both mother and child.

"VIII. A case occurred to Gensoul, in which the feet presented. The mother died in consequence of rupture of the tumour and extravasation of its contents into the abdominal cavity. The fate of the child is not mentioned.

"IX. In one case which terminated fatally, Denman perforated.

"X Ramsbotham and Coutouly extracted the child by the crotchet, and in both instances saved the mothers.

"XI. Coutouly, Meyer, and Gensoul, performed the Cæsarian section. In each instance the woman died. One of the infants was saved, and it is not stated what became of the others." (pp. 223-4 )

The sixth chapter treats of the *different forms of hernia,* which present a mechanical obstacle to labour, and with that the work concludes; nor does the last chapter show any sign of flagging in that diligence with which the author set out. We most cordially recommend the book to all who are interested in the obstetric art: to every lecturer it will be indispensable.

